# Complete Genome Sequence of a *Rhodococcus* Species Isolated from the Winter Skate *Leucoraja ocellata*

**DOI:** 10.1128/genomeA.00918-16

**Published:** 2016-09-01

**Authors:** Julia Wiens, Ryan Ho, Dinesh Fernando, Ayush Kumar, Peter C. Loewen, Ann Karen C. Brassinga, W. Gary Anderson

**Affiliations:** a Department of Biological Sciences, Faculty of Science, University of Manitoba, Winnipeg, Manitoba, Canada; b Department of Microbiology, Faculty of Science, University of Manitoba, Winnipeg, Manitoba, Canada

## Abstract

We report here a genome sequence for *Rhodococcus* sp. isolate UM008 isolated from the renal/interrenal tissue of the winter skate *Leucoraja ocellata*. Genome sequence analysis suggests that *Rhodococcus* bacteria may act in a novel mutualistic relationship with their elasmobranch host, serving as biocatalysts in the steroidogenic pathway of 1α-hydroxycorticosterone.

## GENOME ANNOUNCEMENT

Elasmobranch fish (sharks, skates, and rays) possess a unique corticosteroid, 1α-hydroxycorticosterone (1α-OH-B), produced in the anatomically distinct interrenal gland (1, 2). The complete biosynthetic pathway remains to be elucidated, particularly with respect to the mechanism and enzyme(s) responsible for α-hydroxylation at C-1 (2). Microbial conversion may be a contributing factor, as the presence of a C_11_ hydroxyl group in the β-configuration results in steric interactions favoring this unique α-configuration at C-1 (1, 3). Microbial hydroxylation of C_19_ steroids at positions C-1 and C-2, as well as microbial 1α-hydroxylation, have been demonstrated elsewhere (4, 5). In this view, we have recently isolated bacteria of the genus *Rhodococcus* from the renal/interrenal tissue of the winter skate *Leucoraja ocellata* (J. Wiens, R. Ho, A. K. C. Brassinga, C. A. Deck, P. J. Walsh, R. N. Ben, K. McClymont, A. N. Evans, and W. G. Anderson, unpublished data). Here, we report the genome sequence of the isolate designated *Rhodococcus* sp. UM008, determined by single-molecule real-time (SMRT) sequencing.

The genome of the *Rhodococcus* isolate was assembled using Pacific Biosciences (PacBio) RS II long reads. Briefly, cultures were grown in tryptic soy broth (Difco), and genomic DNA was extracted using the UltraClean microbial DNA isolation kit (Mo Bio Laboratories), modifying the manufacturer’s direction with the addition of incubation with mutanolysin (Sigma-Aldrich) to digest the cell wall (6), in accordance with the established protocol (7). A sequencing-ready PacBio RS II library with 15-kb inserts was prepared at the Génome Québec facility (Montreal, Québec, Canada) and sequenced using C2 chemistry on 3 single-molecule real-time (SMRT) cells, with 180-min movie collection. The combined data consisted of 205,395 reads, with an *N*_50_ size of 9.4 kb. The continuous long reads (CLR) were assembled *de novo* with the PacBio SMRT Analysis software (version 2.3) using the HGAP protocol (8), followed by polishing with Quiver. The NCBI Prokaryotic Genome Annotation Pipeline (9) was then used for functional genome annotation. The chromosome resolved to a single 6,570,200-bp contig with a G+C content of 62.35%. Functional genome annotation with the NCBI Prokaryotic Genome Annotation Pipeline (9) identified 6,050 genes, 15 rRNAs, 53 tRNAs, and 1 noncoding RNA. The chromosome is similar in size, content, and organization to *Rhodococcus erythropolis* PR4 (10). The genome also included three separate contigs representing distinct plasmids.

Genome sequence analysis has identified enzymes orthologous to those characterized in steroid-catabolizing *Actinobacteria* (11), indicating that intracellular *Rhodococcus* bacteria may be involved in the biosynthesis of corticosteroids. We suggest that *Rhodococcus* bacteria may act in a novel mutualistic relationship with their elasmobranch host, serving as biocatalysts in the steroidogenic pathway of 1α-OH-B.

### Accession number(s).

This genome project has been deposited in GenBank under accession numbers CP012749 (chromosome), CP015203 (plasmid 1), CP015204 (plasmid 2), and CP015205 (plasmid 3).
